# Advanced Raman Spectroscopy Detection of Oxidative Damage in Nucleic Acid Bases: Probing Chemical Changes and Intermolecular Interactions in Guanosine at Ultralow Concentration

**DOI:** 10.1021/acs.analchem.1c01049

**Published:** 2021-07-29

**Authors:** Francesca Ripanti, Claudia Fasolato, Flavia Mazzarda, Simonetta Palleschi, Marina Ceccarini, Chunchun Li, Margherita Bignami, Enrico Bodo, Steven E.J. Bell, Filomena Mazzei, Paolo Postorino

**Affiliations:** † Department of Physics, Sapienza University of Rome, P.le A. Moro 5, Rome, Italy; ‡ Department of Physics and Geology, University of Perugia, Via Alessandro Pascoli, Perugia, Italy; § Department of Environment & Health, Istituto Superiore di Sanità, Viale Regina Elena 299, Rome, Italy; ∥ National Centre for Rare Diseases, Istituto Superiore di Sanità, Viale Regina Elena 299, Rome, Italy; ⊥ School of Chemistry and Chemical Engineering, Queen’s University of Belfast, Stranmillis Road, Belfast, Northern Ireland; # Department of Chemistry, Sapienza University of Rome, P.le A. Moro, 5, Rome, Italy

## Abstract

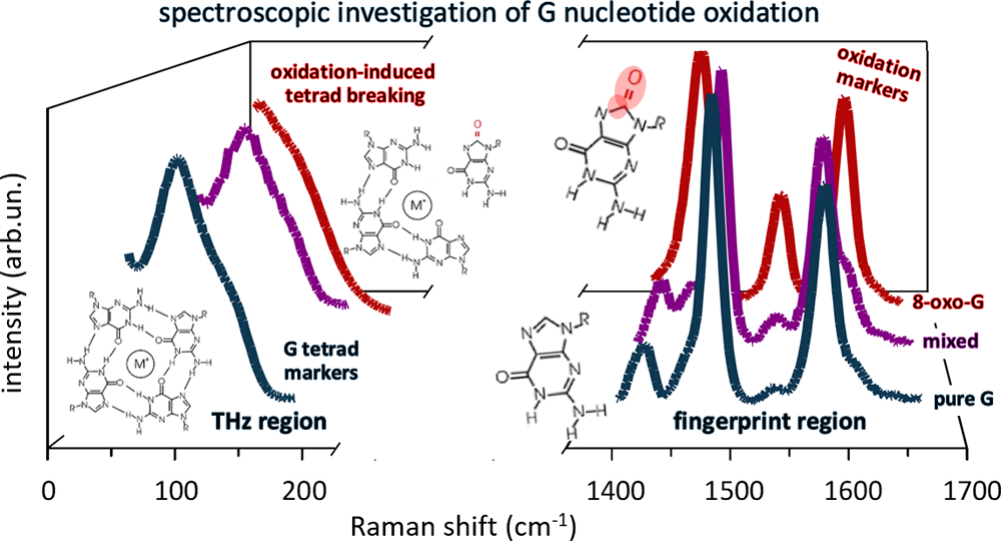

DNA/RNA synthesis precursors are especially vulnerable to damage induced by reactive oxygen species occurring following oxidative stress. Guanosine triphosphates are the prevalent oxidized nucleotides, which can be misincorporated during replication, leading to mutations and cell death. Here, we present a novel method based on micro-Raman spectroscopy, combined with *ab initio* calculations, for the identification, detection, and quantification of oxidized nucleotides at low concentration. We also show that the Raman signature in the terahertz spectral range (<100 cm^–1^) contains information on the intermolecular assembly of guanine in tetrads, which allows us to further boost the oxidative damage detection limit. Eventually, we provide evidence that similar analyses can be carried out on samples in very small volumes at very low concentrations by exploiting the high sensitivity of surface-enhanced Raman scattering combined with properly designed superhydrophobic substrates. These results pave the way for employing such advanced spectroscopic methods for quantitatively sensing the oxidative damage of nucleotides in the cell.

## Introduction

Reactive oxygen species (ROS), such as peroxides, superoxides, and hydroxyl radicals, constitute a major source of damage to cellular components as lipids, proteins, and nucleic acids. Damage occurs when an imbalance among ROS levels and cell antioxidant and repair capability is present, a circumstance that is generally termed oxidative stress.^1−3^ ROS can interact with DNA and RNA molecules, resulting in modification of nitrogen bases,^4,5^ single and double breaks,^6,7^ abasic sites,^8−10^ and DNA/RNA–protein cross-links.^11,12^ Among such ROS-induced harmful effects, DNA/RNA base oxidation is the most frequent, producing more than 20 different types of oxidative damage.^13,14^ Due to its low ionization potential,^14,15^ the guanine base is the most susceptible to oxidation, most commonly resulting in 8-oxo-7,8-dihydro(-2′-deoxy)guanosine, termed 8-oxo-(d)G, in RNA (DNA). 8-oxo-dG and related compounds differ from the non-oxidized counterparts by the presence of a double C=O bond in position 8 of the aromatic ring, instead of a C–H bond. During DNA replication, 8-oxo-dG can pair with adenine instead of the canonical cytosine^5,15^ resulting in GC → TA transversion.^16^ Similar ROS-induced modifications are also found in 8-oxo-deoxyadenosine (8-oxo-dA).^17^ Several lines of evidence indicate that deoxyribonucleoside triphosphates (dNTPs) are relevant targets for oxidation in the nucleotide pool, mainly producing 8-oxo-7,8-dihydro-deoxyguanosine triphosphate (8-oxo-dGTP). Indeed, dNTPs are 13,000-fold more prone to oxidation than bases embedded in DNA,^15^ and the incorporation of oxidized dNTPs in the genome can result in mutagenic DNA damage.^18^ Oxidation can also occur at the level of ribonucleoside triphosphates (NTPs), which are present in large excess over dNTPs in the nucleotide pool.^19−21^

There is a great interest in identifying oxidized DNA and RNA lesions as biomarkers of oxidative stress, particularly in the case of isolated bases, whatever the origin of nucleic acid damage is, that is, direct or mediated by the incorporation of modified precursors. Currently, the typical methods for detecting base modifications include single-cell gel electrophoresis assays,^22−24^ high-performance liquid chromatography (HPLC) coupled with mass spectroscopy or electrochemical detection,^25−27^ and fluorescence staining techniques.^28−30^ All these methods require multiple-step sample preparation and the use of chemicals that may induce additional modifications to the base or interfere with the detection of the modified DNA/RNA. The need for robust, streamlined methods for tracing chemically modified DNA/RNA bases is an important goal for research, mainly in view of future clinical diagnostic applications and translational impact.

In order to address these limitations, here, we propose an approach to detect and quantify the oxidized purine bases through micro-Raman spectroscopy, with a particular focus on guanine. Raman spectroscopy is a non-invasive tool for biological diagnostics, providing direct information on the chemical composition and conformation of biomolecules without the use of fixing agents or fluorescent probes. It has been extensively employed to characterize the chemical structure of DNA nucleotides,^31−34^ as well as to reveal changes in the structure of DNA, such as the formation of oxidative products.^35−37^ Here, by combining Raman data with *ab initio* calculations, we demonstrate the quantitative detection of oxidatively damaged dNTPs and NTPs at low concentration in standard solutions. We also show that the (d)GTP Raman signature in the terahertz spectral range (<100 cm^–1^) contains information on their intermolecular assembly, which can be used to further boost the oxidative damage detection limit. Furthermore, we provide evidence that similar analyses can be conducted on samples in very small volumes and/or at very low concentrations, that is, under conditions verified in cellular extracts, by exploiting the high sensitivity of surface-enhanced Raman scattering (SERS),^38−40^ combined with properly designed superhydrophobic substrates.^41,42^ SERS spectroscopy is based on the enhancement of the Raman scattering signal from a specific analyte either adsorbed or placed in close proximity to a nanostructured noble metal surface. This occurs because the energy of the laser used for Raman excitation, normally in the visible spectral range, is in resonance with the collective excitation of the free electrons (localized surface plasmon resonance) in the metal nanostructures. When the analyte is chemically bound to the nanostructure, a further enhancement occurs due to charge transfer. The SERS detection of specific molecules at ultralow concentration is widely demonstrated in the literature,^39,43,44^ and SERS has been successfully coupled with superhydrophobic substrates for the spectroscopic analysis on microvolume samples.^42,45,46^ Here, we report a novel SERS strategy to quantitatively detect oxidative damage in sub-microliter nucleotide pool solutions, exploiting properly designed and functionalized superhydrophobic needles. Our measurements, indeed, match the detection limits suitable for the biologically analyses.^27^ We believe that these results represent a promising starting point for the development of advanced spectroscopic assays to detect the presence of oxidized nucleotides in cellular dNTP/NTP pools. In principle, this type of investigation might be successfully combined with separative purification techniques, allowing to envision the translation of this approach to clinical applications.

## Experimental Section

### Materials

dGTP, 8-oxo-dGTP, 8-oxo-GTP, and 8-oxo-dATP were purchased from Jena Biosciences GmbH (Jena, Germany), GTP was purchased from Promega (Madison, WI, United States), dATP, deoxyguanosine (dG), and 8-oxo-dG were purchased from Sigma-Aldrich (St. Louis, MO, United States). All the nucleotides and nucleosides were in the stable sodic salt form.

### Mixture Preparation

Using 1 mM nucleotide standard solutions in DNAse-free water, 8-oxo-dGTP and 8-oxo-GTP were diluted at different relative concentrations in dGTP and GTP solution, respectively, ranging from 16 to 0%. Higher 8-oxo-dGTP molar fractions were not considered since such high values have been not revealed in biological samples.^27^ Following this method, mixtures of dATP and 8-oxo-dATP at different relative concentrations were also prepared. Since, to the best of our knowledge, no data are available on the quantification of oxidized adenosine in biological samples, mixtures were prepared at varying oxidized/non-oxidized nucleotide molar ratios in a larger range (50–0%). Measurable samples for Raman spectroscopy were obtained by drop-casting a microvolume (5 μL) of the aqueous solution under examination on a flat gold substrate. We stress that the starting concentration (1 mM) of the nucleotides plays a minor role considering that the Raman spectra were acquired on dried samples (15 min drying under ambient conditions). To obtain measurable samples for SERS experiments, the analyte was mixed with a solution of hydroxylamine-reduced silver nanoparticles, and a 1 μL droplet was deposited on the tip of the superhydrophobic wire and measured thereon.

### Superhydrophobic Substrate Fabrication

The superhydrophobic supports were realized using galvanic deposition on copper wires. The wires (400 μm diameter) were immersed in 0.01 M AgNO_3_ aqueous solution for 1 min, which gave a matt black-textured silver surface coating, and dried. The metal-coated wire was then placed into a 0.01 M solution of a polyfluorinate surface modifier such as 3,3,4,4,5,5,6,6,7,7,8,8,9,9,10,10,10-heptadecafluoro-1-decanethiol in dichloromethane for 2 min and dried. Then, the superhydrophobic coated wire was cut using a sharp scalpel to expose bare copper, which would act as the hydrophilic tip and hold aqueous samples.^41,42^ As a droplet dispenser, we used a gas chromatography syringe, whose needle was given a superhydrophobic coating, so that the dispensed volume readily detached from the needle when it was placed in contact with the tip of the wire support. The syringe needle was coated by first electrodepositing a copper layer at 1.5 V in a simple cell containing CuSO_4_ (1 M) acidified with H_2_SO_4_ and a clean copper foil counter electrode. The copper surface of the syringe was then coated with electroless deposited silver and polyfluorothiol, following the same protocol used for covering the copper wire. This coated syringe allows sub-microliter volume liquid samples to be easily transferred on the support tip. When the droplet was brought into contact with the hydrophilic region of the superhydrophobic wire and the needle, the force holding the droplet to the syringe was less than that holding it to the support tip. A schematic representation of the fabricated superhydrophobic copper needles is shown in Figure S1 of Supporting Information.

### Silver Nanoparticle Preparation

The hydroxylamine-reduced silver colloid was prepared using a well-established protocol.^39,47^ Briefly, 5 mL of NaOH (0.1 M) was added to 5 mL of aqueous hydroxylamine hydrochloride (6 mM); then, the whole mixture was added to 90 mL of aqueous AgNO_3_ (0.1 mM) with stirring. The colloid formed spontaneously and was left stirring for about 20 min before use.

### Micro-Raman Spectroscopy

Raman measurements were carried out using a Horiba HR-Evolution microspectrometer in backscattering geometry, equipped with a He–Ne laser, λ = 632.8 nm and 25 mW output power (≈10 mW at the sample surface, incident flux Φ = 1.9 × 10^3^ mW/cm^2^). The elastically scattered light was removed by a state-of-the-art optical filtering device based on three BragGrate notch filters,^48^ which also allows us to collect Raman spectra at very low frequencies (down to 10 cm^–1^ from the laser line). The detector was a Peltier-cooled charge-coupled device (CCD) and the resolution was better than 3 cm^–1^ thanks to a 600 grooves/mm grating with 800 mm focal length. The spectrometer was coupled with a confocal microscope supplied with a set of interchangeable objectives with long working distances and different magnifications (100× - 0.80 NA was used for the present experiment). Further details on the experimental apparatus can be found in ref (49). Raman spectra were acquired for 5–10 min and preprocessed with LabSpec software (polynomial baseline subtraction in the fingerprint region, linear background in the low-frequency range) and analyzed with Origin Lab code.

### SERS Spectroscopy

Measurements on needles were performed using PerkinElmer RamanMicro 200 apparatus, consisting of a 785 nm external cavity diode laser, outputting 90 mW *via* fiber optic cable to an Olympus BX51 Reflected Illumination microscope, which is equipped with 10×, 20×, and 40× objective lens with different numerical apertures and laser spot sizes. The samples were supported on a manually operated standard microscope stage. The scattered light was collected at 180° through the objective lens and was passed down a separate collection fiber toward the spectrograph, which was based on a Czerny–Turner design. The CCD detector was an electrically cooled Andor DV 420 OE and operated at −50 °C. The instrument had a fixed resolution of 8 cm^–1^ and spectra were collected over the 115–3200 cm^–1^ range. For these experiments, 10× objective (0.25 N.A. and 0.1 mm spot diameter) was used and laser power was set to 30% (incident flux Φ = 2.5 × 10^4^ mW/cm^2^, higher power would burn the samples). Accumulation times were usually varied depending on the sample (10–30 s for single spectrum).

Diluted dG and 8-oxo-dG were mixed with the nanoparticle solution in a 1:10 volume ratio, using MgSO_4_ as an aggregating agent. The final concentrations of the mixtures were in the 10^–4^ to 10^–7^ M range. SERS spectra were analyzed using the partial least squares (PLS) regression through MATLAB software. The spectral range between 400 and 1800 cm^–1^ was chosen since it contains the main bands of dG and 8-oxo-dG (and relative compounds), excluding the characteristic peak of the colloid (around 240 cm^–1^, data not shown) and spectral artifacts. Prior to analysis, unit vector normalization was performed, and three components were adopted for PLS regression.

### *Ab Initio* Calculations

Calculations were performed in the gas phase using the neutral structure corresponding to the bisodic salt of the monoprotonated triphosphate anion for both the dGTP and the 8-oxo-dGTP. Given the large conformational flexibility of such molecules, the conformational space was explored by means of molecular dynamics (MD). In particular, three short independent MD trajectories of 5 ps were recorded using the DFTB + package^50^ with SCC charges,^51^ dispersion interactions,^52^ and the 3ob parameter set. From each trajectory, a set of three structures were extracted for a total of 18 structures. These structures were then optimized at the B3LYP/6-31G* level using the G16 package.^53^ We then selected the seven optimized structures with the lowest energy (all structures turned out to lie within 10 kcal/mol with respect to the lowest energy one) for which we computed harmonic frequencies and Raman activities. The harmonic frequencies were scaled by 0.985 to account for anharmonicity (two of these structures are reported in Figure S2 in Supporting Information).

## Results

### Raman Spectra of Purine Deoxy-Nucleoside Triphosphates

#### Analysis of 8-Oxo-dGTP/dGTP

**Figure 1:**
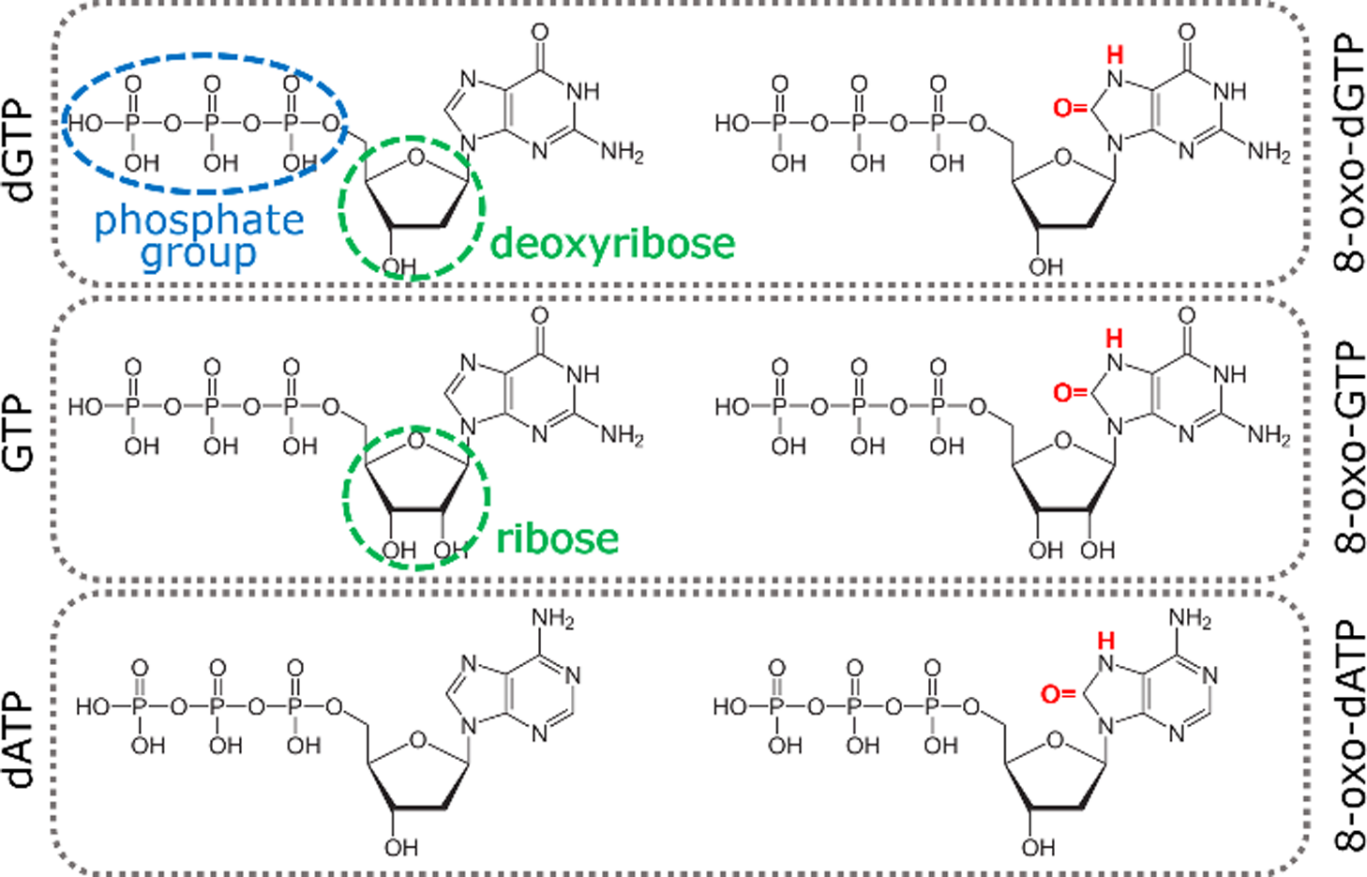
Molecular structures of the analyzed nucleoside triphosphates (left) and their oxidized counterparts (right). From the top: deoxyguanosine triphosphate, guanosine triphosphate, and deoxyadenosine triphosphate. The different sugar component is highlighted in green, while the oxidative damage is marked in red.

A combined theoretical and experimental analysis allowed us to precisely characterize the different vibrational modes of the oxidized and non-oxidized nucleotides. Indeed, the different molecular structures of 8-oxo-dGTP and dGTP (Figure 1) result in different intramolecular electronic distributions, yielding a significantly different Raman response in the fingerprint region (900–1700 cm^–1^), where the spectral bands associated to vibrations within the molecule are present.

**Figure 2:**
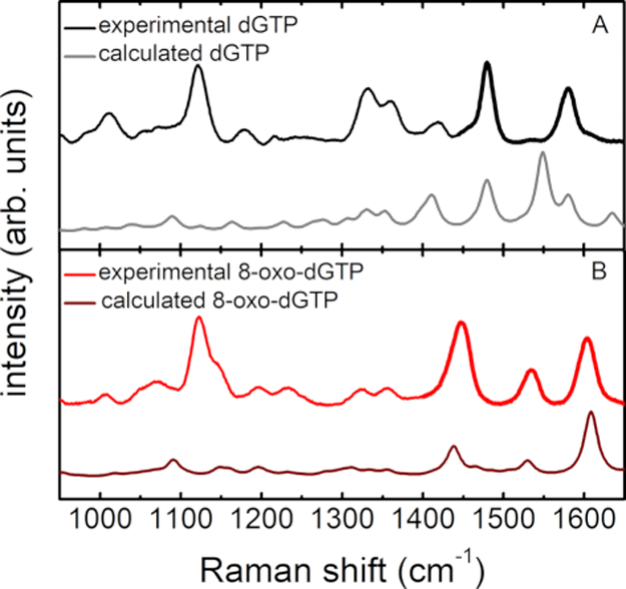
Comparison of experimental and calculated Raman spectra: (A) dGTP and (B) 8-oxo-dGTP. The spectral regions for the quantitative analysis are marked in bold.

To identify the vibrational modes associated to the presence of the extra oxygen in 8-oxo-dGTP, density functional theory (DFT) calculations were carried out. A comparison between calculated and measured spectra for both the dGTP and 8-oxo-dGTP pure samples is reported in Figure 2. The theoretical calculations are consistent with the literature and allowed us to assign the main spectral bands.

Theoretical and experimental data show a remarkable agreement, despite some differences in the frequency values and relative intensities being present, mainly in the case of the dGTP sample. Notably, in the experimental spectra, a strong band at ν_PO_ = 1123 cm^–1^ is clearly detectable. According to our calculations and literature data,^39^ this band can be ascribed to the PO_4_^3-^ phosphate backbone stretching vibration and thus be used for spectra normalization.

The main effect of oxidation is easily recognizable in the 1400–1650 cm^–1^ spectral range (Figure 2). The 8-oxo-dGTP spectrum (panel B) shows three well-defined and separated bands, whereas in the dGTP spectrum (panel A) only two peaks are present. According to our DFT calculations on dGTP, the band at 1485 cm^–1^ is ascribed to the 7N–8C and that at 1575 cm^–1^ to the 3N–4C stretching modes. In 8-oxo-dGTP, the band at 1445 cm^–1^ is associated to the 7N–H bending vibration, the one at 1535 cm^–1^ to the 7N–8C stretching mode of the aromatic ring, and that at 1607 cm^–1^ to the 5C–7N stretching vibration. These data are in good agreement with previous theoretical^54−56^ and experimental^36,37,57^ work.

**Figure 3:**
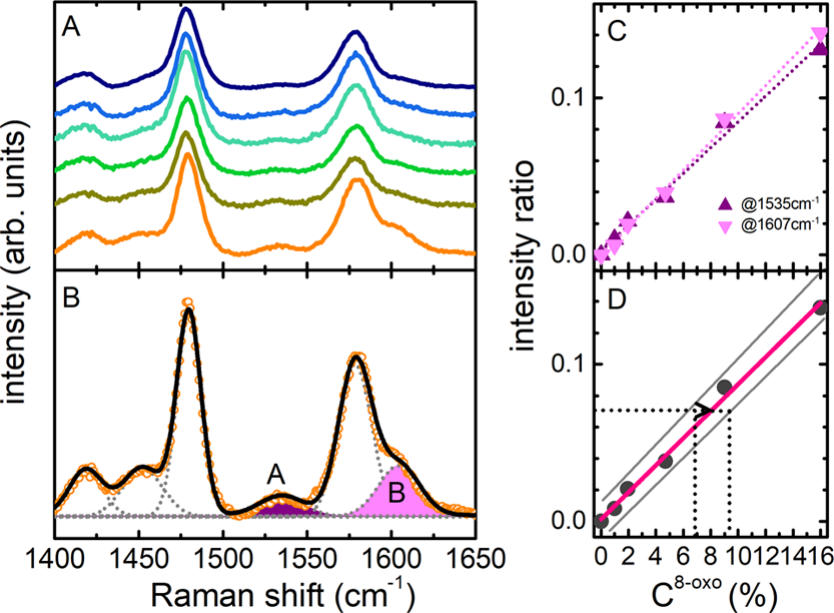
(A) Raman spectra in the fingerprint region of oxidized and non oxidized dGTP mixtures at different 8-oxo-dGTP/dGTP relative concentrations, from 0% (blue) to 16% (orange); (B) representative fitting deconvolution at *C*^8–oxo^ = 16%: hatched areas identify the peaks of 8-oxo-dGTP used for the quantitative analysis; (C) normalized integrated peak intensity (“intensity ratio”) as a function of 8-oxo-dGTP concentration for A (1535 cm^–1^, purple) and B (1607 cm^–1^, magenta) peaks, and linear fit of the data as described in text, with *m*_A_ = 0.008 ± 0.001 and *m*_B_ = 0.009 ± 0.001; (D) calibration curve obtained as the average of the two peak intensities. The 8-oxo-dGTP/dGTP relative concentration and its uncertainty are inferred from the measured intensity (see text for details).

Raman spectra of the mixtures are reported in Figure 3A. Deconvolution of the spectra with six Gaussian curves was carried out in the 1400–1650 cm^–1^ spectral region. The agreement between the fitting curve and the experimental data is excellent for any of the measured mixtures, as shown for a representative sample (*C*^8–oxo^ = 16%) in Figure 3B. In the normalized spectra, the intensity of the two 8-oxo-dGTP bands at ν_A_ = 1535 cm^–1^ and ν_B_ = 1607 cm^–1^, respectively (colored curves in Figure 3B), measures the oxidized base content in the mixture. The Raman intensity *versus* relative concentration trend, derived from fitting, is described by the linear function:
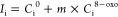
where i = A, B identifies the vibrational peak with intensity *I*_i_, *C*^8–oxo^ is the 8-oxo-dGTP relative concentration in the mixture, and *C*^0^ is the intercept at *C*^8–oxo^ = 0, that is, the value obtained from the fitting procedure for A and B peaks on the dGTP spectrum. By a close inspection of Figure 3A, a weak spectral contribution at the characteristic 8-oxo-dGTP frequencies can be observed in the spectrum of the dGTP alone: this might be ascribed to the accidental presence of a *C*^0^ concentration of 8-oxo-dGTP in the dGTP sample. The *I*_i_ data retrieved from fitting of the mixture spectra, subtracted by the contribution corresponding to *C*^0^ (as reported in Figure S3), are shown in Figure 3C along with the *I*_i_*versusC*_i_^8–oxo^ calibration curves for A and B bands (intercepts = 0 by construction). The calibration lines for A and B have the same slope within uncertainties, confirming that the analysis can be considered as a reliable measurement of the relative 8-oxo-dGTP/dGTP content. The average calibration curve is shown in Figure 3D, with nominal error estimated from the experimental data deviations. Again, by construction, the error is 0 for the dGTP alone (*C*^8–oxo^ = 0). The calibration curve is easily used for assessing the unknown oxidized nucleotide content in a mixture, as sketched in Figure 3D. The proposed protocol yields a detection limit of 1% in terms of relative 8-oxo-dGTP/dGTP concentration, which is comparable to quantification data obtained by Pursell and co-workers^27^ using HPLC with electrochemical detection in mitochondrial extracts.

**Figure 4:**
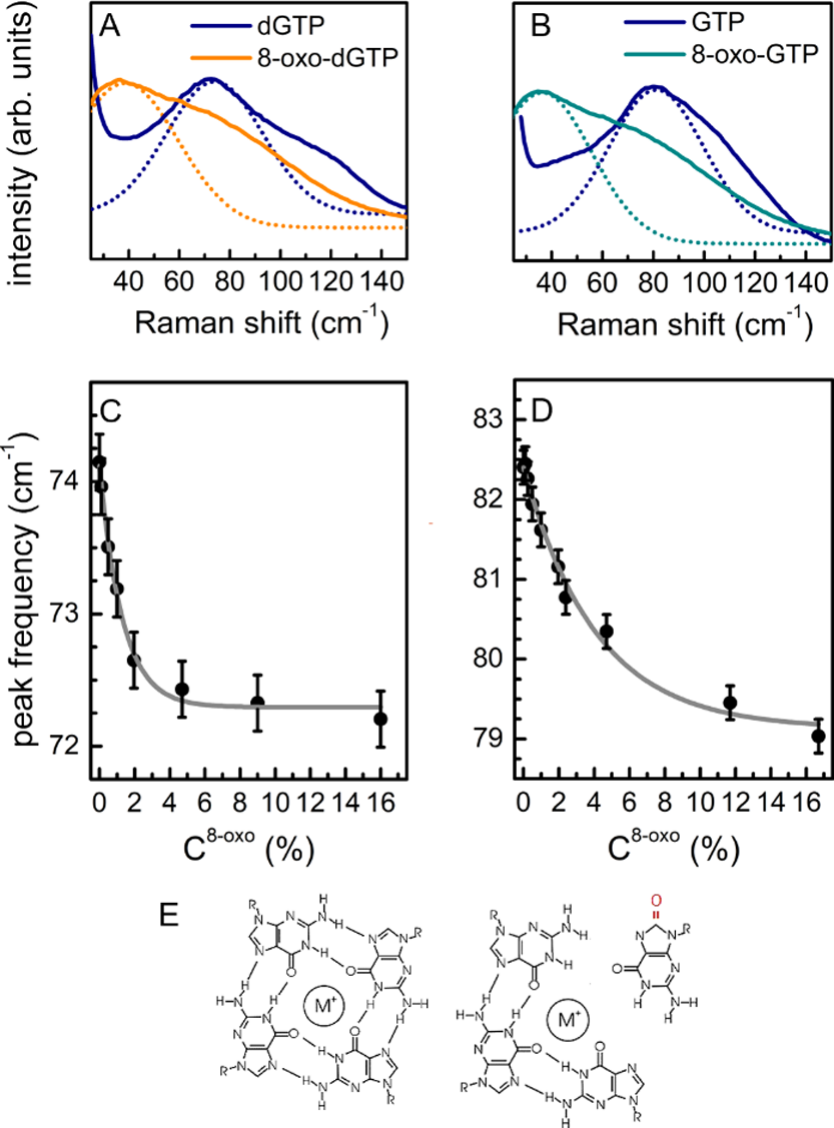
(A) THz Raman spectra of dGTP and 8-oxo-dGTP and the main component of the Gaussian fitting deconvolution; (B) same for GTP and 8-oxo-GTP; (C) frequency of the peak in (A) in 8-oxo-dGTP/dGTP mixtures as a function of the *C*^8–oxo^ relative concentration; and (D) same for 8-oxo-GTP/GTP. (E) Left: schematic representation of a G-tetrad. Right: sketch of the hindered tetrad formation due to the presence of 8-oxo-G: we speculate that some of the H bonds constructing the tetrad cannot form, preventing its construction.

### Analysis of 8-Oxo-dATP/dATP

In order to verify whether a similar method is also suitable for detecting other damaged nucleotides, we characterized the spectroscopic response of 8-oxo-2′-deoxyadenosine-5′-triphosphate (8-oxo-dATP) and repeated the same analysis on oxidized/non-oxidized adenosine mixtures. 8-oxo-dATP differs from its non-oxidized counterpart (dATP) by the presence of an oxygen atom in position 8 of the aromatic ring and of a hydrogen atom bound to the nitrogen in position 7 (Figure 1).^17,58^ The Raman spectrum of oxidized nucleotide significantly differs from the non-oxidized one, although a well-detectable and isolated peak ascribed to the oxidation is not easily identified. The comparison of dATP and 8-oxo-dATP Raman spectra in the fingerprint region is shown in Supporting Information (Figure S4A). Based on our analysis, the best oxidation marker is the spectral band around 620 cm^–1^, ascribed to the C5–N7–C8 squeezing vibration,^59,60^ which is present almost exclusively in the 8-oxo-dATP spectrum. Following the same procedure as mentioned above, mixtures of 8-oxo-dATP/dATP at different relative concentrations were prepared and the corresponding Raman spectra were collected. The spectral weight of the 620 cm^–1^ band was evaluated through an accurate fitting procedure and the normalized integrated areas as a function of the 8-oxo-dATP content are reported in Figure S4B. A clear linear dependence on the concentration was observed, providing a safe 5% oxidized nucleotide detection limit. The result is less striking compared to dGTP, an aspect well-explained considering the spectral range of the selected 8-oxo-dATP marker. In the case of 8-oxo-dATP, a single isolated peak was not available for the analysis and a dATP-associated spectral background produces some noise in the quantitative measurements. Nevertheless, the protocol yields results that are consistent with the case of guanine compounds, thus allowing for the quantification of the oxidation level also for adenine samples.

### Raman Spectra of NTPs

#### Analysis of GTP/8-Oxo-GTP

Since our general aim is to propose sensitive methods for the quantification of oxidized nucleotides in cellular pools, which are dominated by the presence of ribonucleotides, we carried out our analysis also on ribonucleotide mixtures, with different relative concentrations of GTP and 8-oxo-GTP. NTPs are characterized by the presence of a ribose sugar unit, which differs from deoxyribose for the presence of an oxygen atom bound to the 2-carbon (Figure 1). The sugar type does not affect the spectroscopic response, at least in the fingerprint spectral range, as suggested in previous work.^61^ The Raman spectra of the single (d)GTP/8-oxo-(d)GTP nucleotides look very similar (Figure S5 in Supporting Information). This result suggests that the quantitative analysis can be extended to the ribonucleotide mixtures.

The spectra of GTP and 8-oxo-GTP samples reproduce the differences observed on the deoxy counterpart with the same spectral markers of oxidation, that is, the peaks at ν_A_ = 1535 cm^–1^ and ν_B_ = 1607 cm^–1^ (Figure S6A in Supporting Information). 8-oxo-GTP/GTP mixtures at different relative concentrations (0–16% range, as in the deoxy case) were spectroscopically analyzed. The same procedure (normalization, subtraction of pure GTP contribution, and linear fitting) yielded the calibration curve reported in Figure S6B, with a slope in agreement with that obtained for the 8-oxo-dGTP/dGTP mixtures. This demonstrates that the Raman signature of oxidation is not affected by the presence of different sugars. For both cases (dGTP and GTP), the oxidative damage detection limit is assessed around the 1% oxidized/non-oxidized relative concentration.

### Monitoring Intermolecular Organization by Terahertz Raman Spectroscopy

At high concentration, guanine compounds are known to form self-ordered tetrad aggregates, usually referred to as G-tetrads or G-quartets, which tend to stack in columnar structures.^62−64^ These structures are made of piles of planar tetramers, stabilized by eight Hoogsteen and Watson–Crick hydrogen bonds (N1–O6 and N2–N7), with the O6 atoms centrally oriented into the ring to form an anionic bipyramidal cage that can coordinate to monovalent cations (*e.g.,* Na^+^ and K^+^). G-tetrads are present also in G-rich DNA sequences, in crucial domains of the genome, and in RNA sequences,^65−68^ due to the folding of DNA/RNA strands. These structures are termed G-quadruplexes. The presence of oxidative damage can interfere with the formation of such intermolecular organization, hindering the biological role. Indeed, the different structure and electronic distribution in oxidized nucleotides produce structural rearrangements at the intermolecular level, modifying the extent and the properties of long-range intermolecular interactions, which are reflected in the THz Raman response. In the past, the characterization of the self-assembled structures has been carried out exploiting nuclear magnetic resonance spectroscopy, scanning electron microscopy, dynamic light scattering, and fingerprint Raman analysis.^61,69−71^ To the best of our knowledge, the low-frequency G-tetrad Raman response has not been reported yet and has the advantage of containing intense and easily monitored markers of tetrad formation.

Exploiting the remarkable performance of volume Bragg filters for ultralow-frequency detection of the Raman signal,^48^ we developed a complementary spectroscopic strategy for oxidative damage quantification, by focusing on the THz Raman response. The spectral features associated with intermolecular organization can be probed in the very low-frequency region, that is, down to 20 cm^–1^. The THz Raman spectra of dGTP and 8-oxo-dGTP are shown in Figure 4A. Both spectra are characterized by a broad structure, differing by a remarkable (>30 cm^–1^) shift in the frequency of the main band. The same occurs in the spectra of GTP and 8-oxo-GTP (Figure 4B). The fitting deconvolutions are shown in Figure S7 in Supporting Information. A gradual frequency shift depending on oxidized/non-oxidized relative concentration is observed in the mixture spectra, as shown in Figure 4C,D for deoxy- and ribonucleotides, respectively. Remarkably, in both cases, the main peak frequency ν_*C*^8–oxo^_ as a function of *C*^8–oxo^ is well-described using an exponential curve:
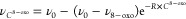
where ν_0_ and ν_8–oxo_ are the asymptotic values for GTP and 8-oxo-GTP, respectively. The fitting provides two different values for the parameter R in the two cases (deoxy- and ribonucleotides), which imply two different decay rates for the exponential curves. This is reasonably ascribed to the role of the sugar in the long-range intermolecular interactions.^61^

Summarizing, we speculate that the observed trend is associated to the tetrad formation process. The low-frequency features (Figure 4A,B) can be considered as collective intermolecular modes and thus be attributed to vibrations associated to the G-tetrad structure.^68^ Notably, a different THz Raman feature is observed in the oxidized samples. Therefore, due to the presence of an O8 atom, 8-oxo-(d)GTP can form different bonds with the neighboring N6, involved in the tetrad H-bonds, hence impeding the aggregation process. Therefore, tetrad structures cannot form in these solutions (Figure 4E). The presence of supramolecular aggregates in this case is still under study and continuous helical structures have been proposed.^72^ This hypothesis explains the large difference between the oxidized and non-oxidized spectra in the THz region. In the case of mixtures, the presence of oxidized molecules interferes with the tetrad formation. Indeed, at low oxidation levels, molecules are mostly ordered in aggregates, while, at increasing oxidation, some of the tetrads are expected to break down or their formation to be inhibited, thus decreasing the number of aggregates. Remarkably, the analysis of intermolecular effects provides a detection limit around 0.2%, therefore boosting by an order of magnitude the sensitivity obtained from the fingerprint Raman analyses.

### Oxidized Nucleoside Detection in Microvolume Samples by SERS

To enable the application of the proposed spectroscopic method to biological samples, we exploited the enhanced sensitivity of SERS spectroscopy. We used superhydrophobic substrates to further reduce the minimum sample volume required for the analysis. Different from the standard strategies for preparing synthetic superhydrophobic materials, including chemical reactions, chemical vapor deposition, nanolithography, electrospinning, layer-by-layer self-assembly, and phase separations,^73,74^ we exploited the alternative method proposed in refs (41)(42), and (45): a cost-effective and time-saving approach based on electroless galvanic deposition of Ag from an aqueous solution onto Cu needles, as explained in the Experimental Section. The hydroxylamine-stabilized Ag colloid was prepared according to a well-established protocol,^39,47^ resulting in a negatively charged colloid. Since (d)GTP and 8-oxo-(d)GTP nucleotides are negatively charged due to the phosphate groups, to favor a better electrostatic sample–colloid interaction, SERS measurements were performed directly on dG and 8-oxo-dG. We made sure that the Raman spectrum of the nucleosides preserved the characteristic spectral features of oxidation.^74^

**Figure 5:**
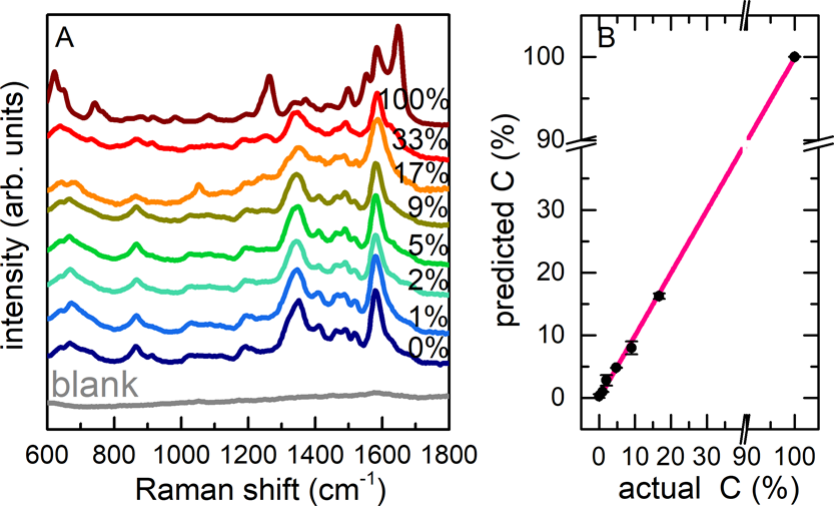
(A) SERS spectra of nucleoside mixtures (10^–4^ M) at varying *C*^8–oxo^ from 0% (solid blue line) to 100% (wine) acquired on superhydrophobic substrates, not corrected by any baseline subtraction. The blank colloid spectrum is reported for comparison (dashed line); (B) multivariate data analysis of the SERS spectra: PLS regression plot of predicted *versus* actual *C*^8–oxo^. We report PLS regression residuals as error bars.

As a proof-of-principle experiment to verify the sensitivity and the reproducibility of the spectra acquired on the needles, dG solutions at decreasing concentration were first analyzed (Figure S8 in Supporting Information). At least down to 10^–7^ M, high-quality spectra were collected. Notably, such an analysis allows us to recognize extremely small amounts of molecules: a 1 μL droplet at 10^–7^ M analyte concentration corresponds to less than a picomole. Test mixtures (10^–4^ M concentration, *C*^8–oxo^ in the range explored by Raman) were prepared and analyzed following the procedure above described, and the obtained SERS spectra are shown in Figure 5A as a function of the relative content of 8-oxo-dG.

Some spectroscopic markers of the presence of 8-oxo-dG can be identified by just looking at the mixture spectra (*e.g.,* the features around 1250 cm^–1^ and in the 1450–1700 cm^–1^ region). However, their quantification is not trivial even by spectral deconvolution because of the intrinsic complexity of SERS spectra.^75−78^ PLS regression resulted as a more successful approach, allowing us to discriminate the spectral features of the oxidized/non-oxidized molecules. PLS analysis is based on a model where a few spectra are employed to create a calibration curve used in quantitative analysis (details provided in the Experimental Section). The model-predicted 8-oxo-dG concentrations are plotted *versus* the actual values in Figure 5B. The data show a linear behavior and an 8-oxo-dG sensitivity less than 2% is reached, in complete agreement with the results of the fingerprint Raman analysis (further details provided in Supporting Information). Moreover, the SERS approach allows for a remarkable reduction of the sample volume and concentration. We remark, in this experiment, mixed samples were prepared at 10^–4^ M, but similar high-quality spectra can be obtained even at much lower concentrations of guanosine alone (Figure S8). The strength of SERS, indeed, allows us to remarkably reduce the total number of probed molecules needed for the discussed investigation. In principle, these findings prove the feasibility of femtomolar 8-oxo-dG detection.

## Conclusions

In conclusion, we developed diverse Raman spectroscopy-based assays for the detection of oxidative damage in a nucleotide pool, mainly exploring the biologically relevant case of oxidized guanine. Compared to other diagnostic methods, Raman spectroscopy is realized by a single-step measurement protocol, rapidly providing chemically specific signatures of the detected molecules. The discussed methods can be extended to other types of oxidative damage to DNA/RNA nucleotides, as demonstrated in the case of adenine.

When looking at the Raman spectra in the fingerprint spectral region, where bands associated to the vibration of specific chemical groups are found, some spectral markers of oxidation can be identified. DFT calculations coupled with Raman spectroscopy allowed the specific assignment of the vibrational modes associated to oxidation. By studying the fingerprint Raman response of artificial nucleotide mixtures, we demonstrated that 8-oxo-(d)G can be revealed in a pool of non-oxidized (d)G down to relative concentrations as low as 1%, which are quantitatively determined by a thorough spectral deconvolution analysis.

Furthermore, THz Raman spectroscopy, which provides information on intermolecular organization, enabled monitoring the formation of tetrad G-aggregates. The characterization of the low-frequency Raman signature associated to the formation of G-tetrads is, to the best of our knowledge, a novel result of the present work that might be exploited for studying G-quadruplex structures for a wide variety of biological applications. Our results on mixtures of oxidized/non-oxidized molecules suggest that the presence of damaged bases in the nucleotide pool hinders the formation of tetrads, resulting in a modified THz Raman response. This can be analyzed for quantitatively assessing the percentage of oxidized nucleotides well below the 1% detection limit.

To further enhance the sensitivity of our analysis, we turned to SERS, which we exploited in combination with superhydrophobic substrates to spectroscopically probe microliter sample volumes. We characterized the SERS signature of (d)G and 8-oxo-(d)G and demonstrated that SERS measurements provide high-quality spectra from μL droplets down to sub-micromolar concentration. The SERS detection limit of 8-oxo-dG in an oxidized/non-oxidized nucleoside pool is around 1%, which is well-sufficient for biomedical applications. Based on these results, we speculate that the detection of oxidized guanosine femtomoles is feasible, paving the way to applications exploiting the ultrasensitive detection of oxidized guanine in cells.

Because of the versatility and simplicity of the procedure, the discussed proof-of-principle results are relevant as a preliminary step toward clinical applications. Translating this approach to the analysis of real cellular extracts might be facilitated by coupling the spectroscopic analysis with a separative technique such as HPLC, to isolate the nucleotide fraction from other components of the cellular extract and chemicals employed for the treatment. The evidence that very small sample volumes are needed for the quantification ensures that the detection limits retrieved in our proof-of-principle analysis are within reach in real applications.

## Supporting Information Available

Further details on calculations and experiments, additional spectral analysis, fit deconvolution, and PLS regression results (PDF)
