# Supplementary material for: Advanced Raman Spectroscopy Detection of Oxidative Damage in Nucleic Acid Bases: Probing Chemical Changes and Intermolecular Interactions in Guanosine at Ultralow Concentration
Source: Anal Chem. 2021 Jul 29;93(31):10825–33. doi: 10.1021/acs.analchem.1c01049 (PMC8382216; doi:10.1021/acs.analchem.1c01049)
Supplement: Supplementary file 1 — ac1c01049_si_001.pdf [file ac1c01049_si_001.pdf]

# SUPPORTING INFORMATION

# Advanced Raman spectroscopy detection of oxidative damage in nucleic acid bases: probing chemical changes and intermolecular interactions in guanosine at ultralow concentration

Francesca Ripanti[1*], Claudia Fasolato[2*], Flavia Mazzarda[1†], Simonetta Palleschi[3], Marina Ceccarini[4], Chunchun Li[5], Margherita Bignami[3], Enrico Bodo[6], Steven E.J. Bell[5], Filomena Mazzei[3], Paolo Postorino[1]

[1] Department of Physics, Sapienza University of Rome, P.le A. Moro 5, Rome, Italy

[2] Department of Physics and Geology, University of Perugia, via Alessandro Pascoli, Perugia, Italy

[3] Department of Environment & Health, Istituto Superiore di Sanità, Viale Regina Elena 299, Rome, Italy

[4] National Centre for Rare Diseases, Istituto Superiore di Sanità, Viale Regina Elena 299, Rome, Italy

[5] School of Chemistry and Chemical Engineering, Queen's University of Belfast, Stranmillis Road, Belfast, Northern Ireland

[6] Department of Chemistry, Sapienza University of Rome, P.le A. Moro, 5, Rome, Italy

† present affiliation: Frank Reidy Research Center for Bioelectrics, Old Dominion University, Norfolk, VA, USA

**Table of content:**


- Experimental details on superhydrophobic substrate fabrication and *ab initio* calculation
- Further details on 8-oxo-dGTP detection and deconvolution analysis
- Raman results on dATP/8-oxo-dATP mixed samples in the fingerprint spectral region
- Effect of ribose and deoxyribose on the dGTP/GTP Raman response
- Raman results of 8-oxo-GTP/GTP mixtures in the fingerprint spectral region
- Details of fitting deconvolution procedure in the low frequency spectral region
- SERS detection limit of guanosine samples with superhydrophobic substrates
- Details of PLS regression analysis on the SERS and Raman spectra of G/8-oxo-G mixtures


**Experimental section: superhydrophobic substrate fabrication**

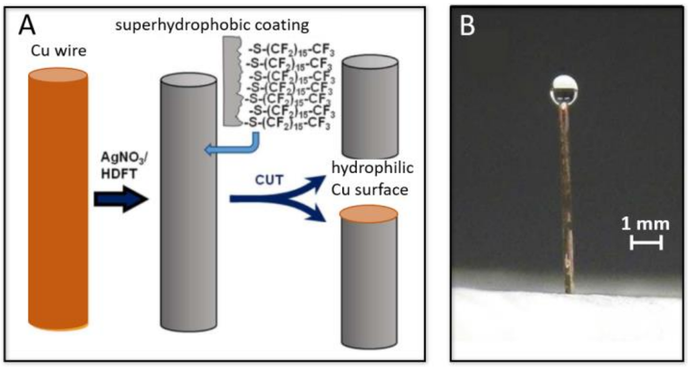


**Fig. S1** (A) Schematic diagram of the experimental procedure for the fabrication of superhydrophobic substrates; (B) photograph of a superhydrophobic needle with a sample droplet on the tip. The volume of the droplet is 1 μL. Adapted with permission from [Cheung, M.; Lee, W.W.Y.; McCracken J.N.; Larmour, I.A.; Brennan, S.; Bell, S.E.J. Raman analysis of dilute aqueous samples by localized evaporation of submicroliter droplets on the tips of superhydrophobic copper wires. Anal. Chem. 2016, 88, 4541–4547]. Copyright © 2016 American Chemical Society.

## Experimental section: *Ab initio* calculation results

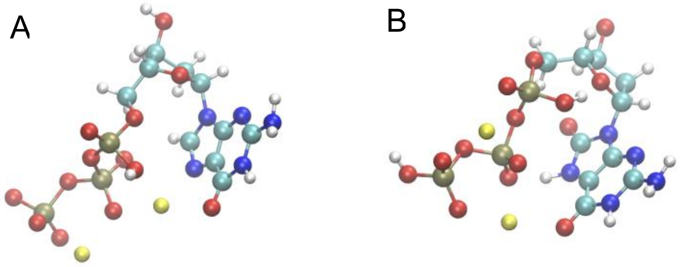


**Fig. S2** Two of the minimum energy structures of the bisodic salts of dGTP (A) and 8-oxo-dGTP (B).

## Raman detection of 8-oxo-dGTP in dGTP mixtures

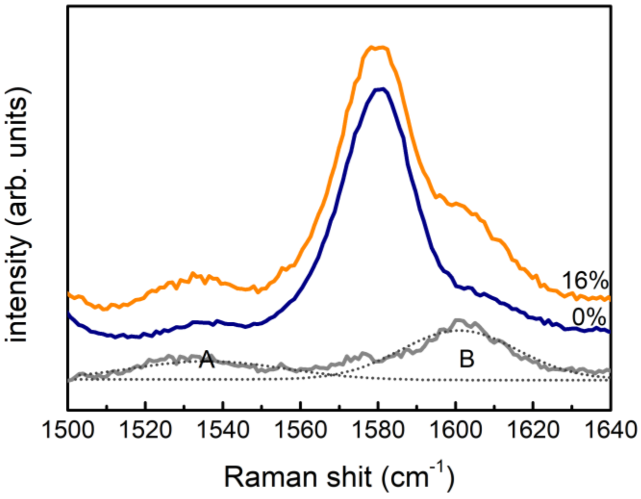


**Fig. S3** Raman spectra of dGTP/8-oxo-dGTP mixture with 16% of oxidized 8-oxo-dGTP (orange line), after the subtraction (gray line) of dGTP (blue line). The subtracted spectrum still shows the markers of oxidation at 1535 $cm^{-1}$ (A band) and 1635 $cm^{-1}$ (B band).

## Raman detection of 8-oxo-dATP in dATP mixtures

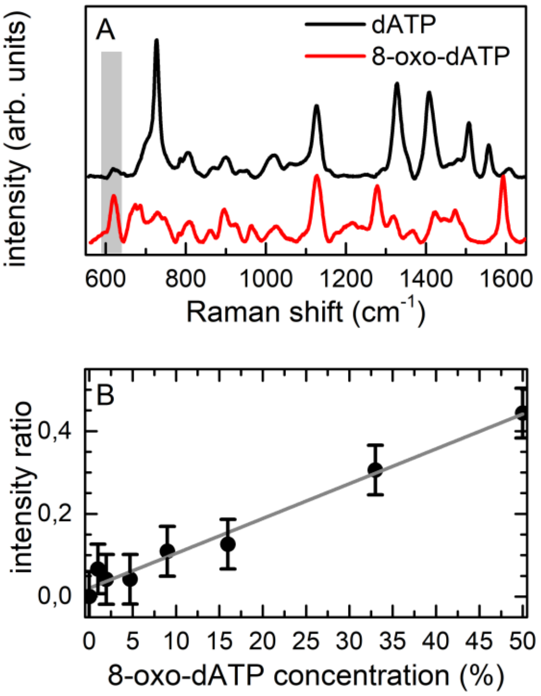


**Fig. S4** (A) Raman spectra of dATP (black line) and 8-oxo-dATP (red line). Peak at 620 $cm^{-1}$, selected as marker of oxidation, is highlighted; (B) integrated intensity of the band at 620 $cm^{-1}$ measured in the mixture spectra as a function of 8-oxo-dATP relative concentration.

## Comparison of oxidized and non-oxidized guanine compounds of DNA and RNA: effect of the different sugar

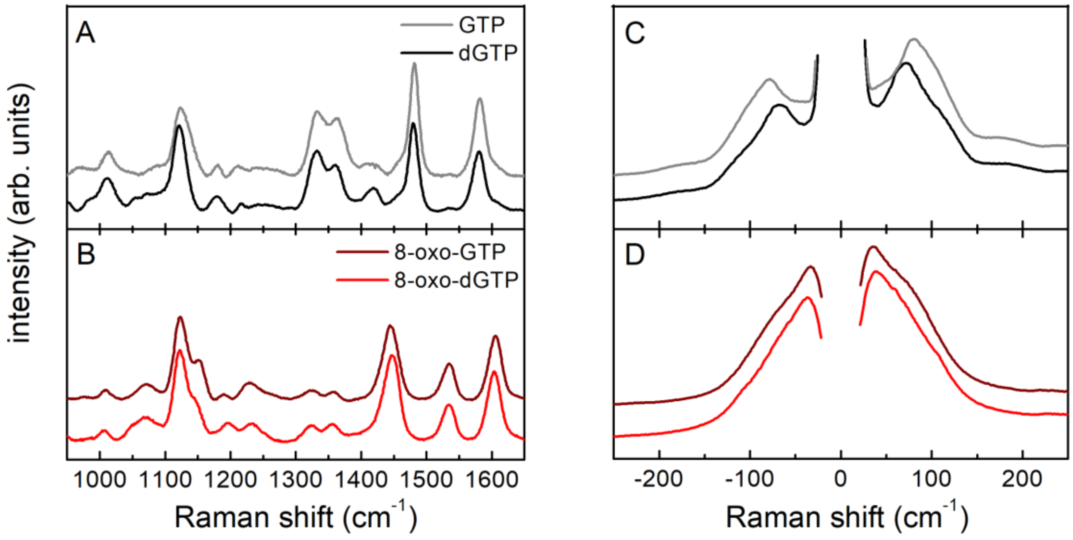


**Fig. S5** (A) Raman spectra of dGTP (black) and GTP (gray) in the fingerprint region; (B) Raman spectra of 8-oxo-dGTP (red) and 8-oxo-GTP (wine) in the fingerprint region; (C) Raman spectra of dGTP (black) and GTP (gray) in the THz range; (D) Raman spectra of 8-oxo-dGTP (red) and 8-oxo-GTP (wine) in the THz range.

## Results of 8-oxo-GTP and GTP mixtures in the fingerprint spectral region

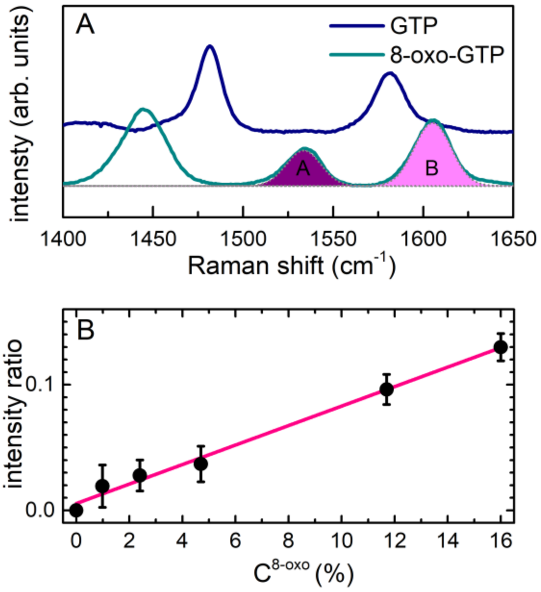


**Fig. S6** (A) Comparison of Raman spectra of GTP (blue, top) and 8-oxo-GTP (dark cyan, bottom) in the 1400-1650 cm$^{-1}$ spectral region. The spectral markers of oxidation, A and B bands at 1535 and 1607 cm$^{-1}$, are fitted with Gaussian curves and evidenced by the colored areas; (B) calibration curve obtained as the average of two integrated intensities (A, B) vs $C^{8-oxo}$. Error bars are estimated by the partial dispersion of repeated measurements. The slope is $m_{ribo}$ = 0.008 ± 0.001.

**Data fitting in the low frequency spectral range**

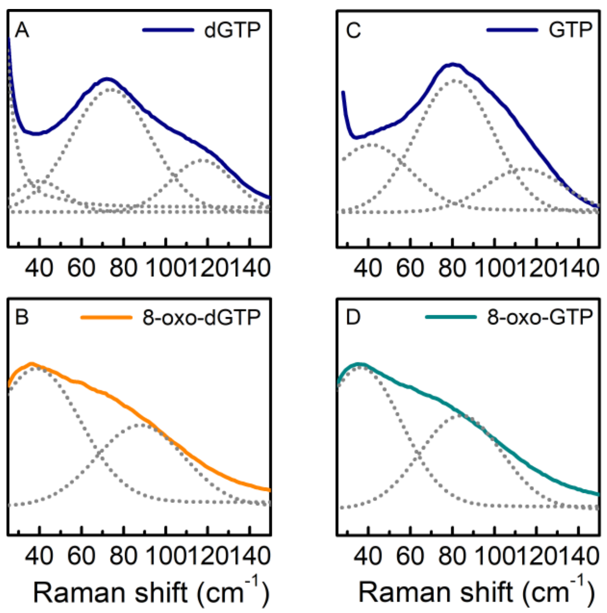


**Fig. S7** Gaussian fitting deconvolution of the THz Raman spectra of: (A) dGTP, (B) 8-oxo-dGTP, (C) GTP, and (D) 8-oxo-dGTP.

**Sensitivity of SERS combined with superhydrophobic substrates**

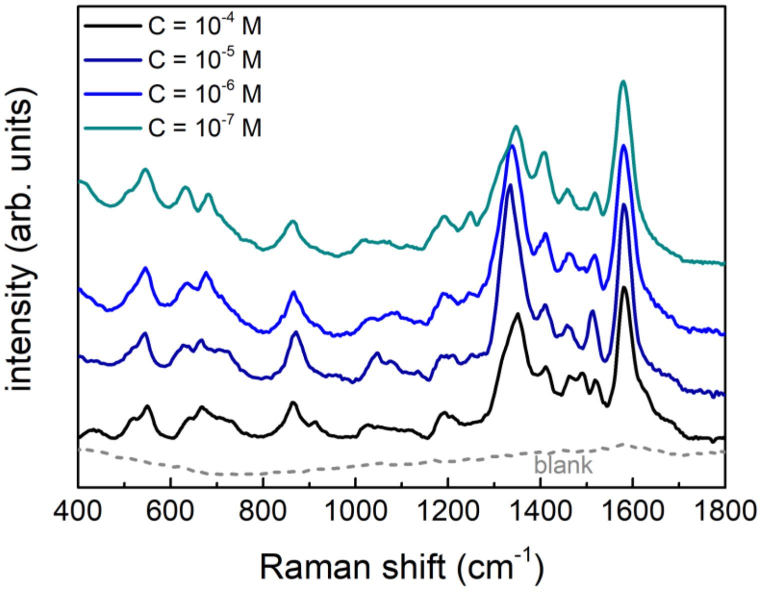


**Fig. S8** Guanosine SERS spectra acquired on the superhydrophobic needle tip at different absolute concentrations of the sample.

## PLS regression results

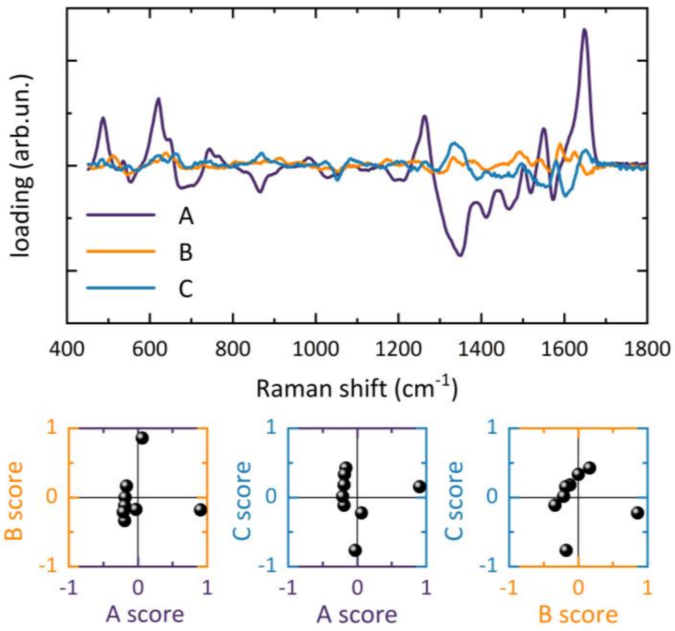


**Fig. S9** Loadings (top) and scores (bottom panels) for the 3 components PLS regression analysis performed on the SERS spectra of nucleoside mixtures at varying $C^{8\text{-oxo}}$ (0%-100%). Loading A shows a positive intensity correspondence of the characteristic peaks of 8-oxo-dG and a negative contribution in the region of the characteristic dG bands (see Fig. 5(A) in the main text). Loadings B and C feature spectral contributions mainly associated to dG. In the score plots, a marked separation between the spectra of pure dG and 8-oxo-dG and the spectra of the mixtures is visible. The predicted concentration is shown in Fig. 5(B) in the main text.

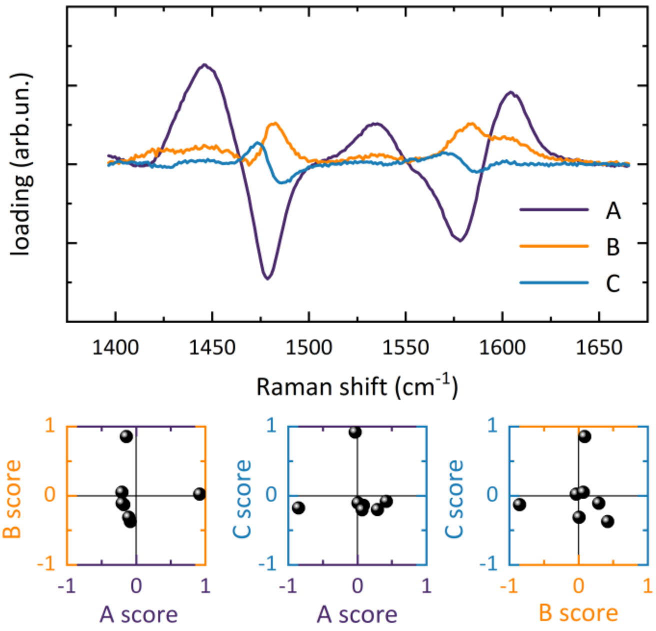


**Fig. S10** Loadings (top) and scores (bottom panels) for the 3 components PLS regression analysis performed on the Raman spectra of G/8-oxo-G mixtures at varying $C^{8\text{-oxo}}$ (0%-100%). Similar to the SERS analysis, loading A shows a positive intensity correspondence of the characteristic peaks of 8-oxo-G and a negative contribution in the region of the characteristic G bands (see Fig. 3(A) in the main text). Loadings B and C feature spectral contributions mainly associated to pristine G. In the score plots, a marked separation between the spectra of pure G and 8-oxo-G and the spectra of the mixtures is visible.

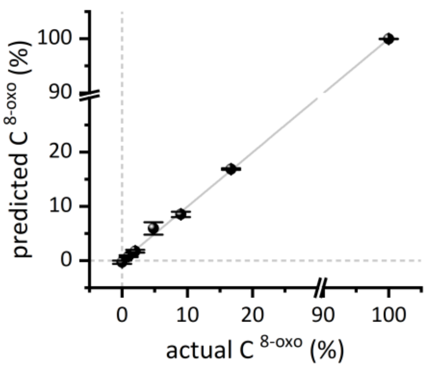


**Fig. S11** Predicted versus actual relative 8-oxo-G concentration ($C^{8\text{-oxo}}$) as estimated by PLS regression applied to the Raman data in Figure 3(A). Error bars are estimated from PLS residuals. As already shown in the case of fitting deconvolution, the excellent quality off the spectra allows a solid determination of $C^{8\text{-oxo}}$ also from PLS regression analysis.
